# Meteorological Register

**Published:** 1845-10-01

**Authors:** 


*Meteorological Register.*—Our readers will observe that the Meteorological Register is omitted in this, and in the last No. of the Journal. This is owing to the unexpected removal of the United States troops, and the abandonment of the military post at this place. Our plan of collecting a connected series of observations, must, for the present, be abandoned.

We have received from Lieut. E. R. Long, M. D., a statistical report of the diseases which have occurred among the U. S. troops stationed here, for for the three past years, which will receive attention in our next number. Other communications have, also, been received, and are on file for publication.

